# “Phone in the Room, Mind on the Roam”: Investigating the Impact of Mobile Phone Presence on Distraction

**DOI:** 10.3390/ejihpe15050074

**Published:** 2025-05-08

**Authors:** Andrea Christodoulou, Petros Roussos

**Affiliations:** Department of Psychology, National and Kapodistrian University of Athens, 15784 Athens, Greece; roussosp@psych.uoa.gr

**Keywords:** nomophobia, attention, mobile phone presence, smartphone distraction

## Abstract

In the digital age, mobile phones significantly impact human cognition and behavior. This experimental study examined the effects of passive mobile phone presence on attentional control in young adults aged 18–25. Participants were randomly assigned to a control (no phone) or an experimental group (phone present). Attention control was measured using the Attention Network Test (ANT). In contrast, smartphone nomophobia and addiction were measured with the Nomophobia Questionnaire (NMP-Q) and the Smartphone Addiction Scale-Short Version (SAS-SV). Contrary to previous literature, the presence of a mobile phone did not significantly distract participants or impair attentional performance. No significant relationship emerged between self-reported levels of distraction or nomophobia and actual attentional performance, although smartphone addiction seemed to have a weak effect on the errors made by those who performed in the presence of their mobile phone. Significant gender differences were found in terms of nomophobia, with women reporting higher levels than men. This study suggests that the relationship between mobile phone presence and attentional processes is more complex than previously hypothesized, bringing the existing literature under further consideration.

## 1. Introduction

In an era characterized by the pervasiveness of digital technology, the prevalence of mobile phones has become a defining feature of contemporary life. These devices have revolutionized everyday life by offering us the ability to access and consume information, communicate, and perform various tasks, changing attitudes, habits, routines, and behaviors (Fortunati, 2023). The investigation of the impact of mobile phones on human cognition and behavior has emerged as a crucial area of research. Alongside their unquestionable usefulness, concerns have been raised about the potential impact of mobile phone use on cognitive processes, with reference to attention (Wilmer et al., 2017). The focus of this research is a profound interest in the impact of passive mobile phone use on attention, particularly among young adults aged 18–25 years. While previous research has concentrated on the effects of active mobile phone use (Stothart et al., 2015; Zheng & Lee, 2016), there persists a notable gap in our comprehension of the subtle yet pivotal influence of mobile phones on attentional control. Despite the pervasiveness of mobile phones in everyday life, there is a paucity of evidence regarding the potential impact of their passive presence as a source of distraction. The present research seeks to form a new perspective on existing literature and provide new insights about attention and how it is mediated by technology. This research’s importance extends the boundaries of the academic field and includes more general social implications since this issue has not been explored before in Greece. As mobile phones continue to shape the web of social interaction and well-being, understanding their cognitive and psychological impact becomes an increasingly important consideration. Moreover, this study’s demographic specificity—targeted at young adults, a demographic Mobile Phone Presence and Attention group highly engrossed in mobile technology—highlights its importance in addressing the evolving challenges raised by digital devices in modern society (Y. K. Lee et al., 2014; S. Lee et al., 2017; Yildirim & Correia, 2015).

### 1.1. Mobile Phone Presence and Attention

In the modern digital age, mobile phones have become ubiquitous, essential tools that have pervaded almost every aspect of our daily lives (Oulasvirta et al., 2012). From communication and access to information to entertainment and productivity, these devices have completely revolutionized how individuals interact with the world around them (Rosen et al., 2013). However, amid their mass adoption, there is a complex interaction of cognitive, behavioral, and social implications that requires a closer examination (Lin et al., 2015).

Despite their practical usefulness, mobile devices have been implicated in various cognitive challenges, particularly in attention (Rosen et al., 2013). The constant stream of notifications and triggers from mobile devices can interrupt attentional processes, leading to difficulty maintaining focus and cognitive performance (Lin et al., 2015). The pervasiveness of online information has led to the tendency for individuals to frequently shift attention and engage in concurrent tasks rather than maintain focus on a single task or activity (Roussos, 2023). Mobile phones are a source of internet applications. The design of various internet applications is engaging, addictive, and distracting, which impairs the ability of individuals to maintain their attention on a cognitive task due to the constant notifications that demand and distract attention (Gazzaley & Rosen, 2016). Interruptions within the phone cause a delay of up to four times the time it takes to complete a primary task (Lui & Wong, 2012). In a task requiring attention, mobile phone notifications disrupt performance similar in scale to active phone use (Thornton et al., 2014). The cognitive demands of mobile phones bring cognitive resources into decline, resulting in decreased efficiency and accuracy in task performance (Clayton et al., 2015).

The relationship between mobile phones and attention is complex, with data indicating that users immediately direct their attention to their smartphone upon receipt of a notification, regardless of whether the device is set to vibrate or silent mode (Chang & Tang, 2015; Pielot et al., 2014; Sahami Shirazi et al., 2014). Studies have demonstrated that smartphone notifications can significantly distract individuals, even when they do not respond. Furthermore, there is evidence that individuals respond more frequently to smartphone-related sounds than to other tones (Stothart et al., 2015). Indeed, several studies have demonstrated that the mere presence of a mobile phone can significantly impact attentional processes, even when the device is not actively in use (Clayton et al., 2015). This phenomenon, known as the mere presence effect, highlights mobile phones’ delicate yet powerful impact on cognitive function (Reinecke et al., 2018).

To comprehend the influence of mobile phones on attention, it is essential to grasp the concept of ‘brain drain’. This concept posits that the cognitive resources of attention are constrained in their capacity for attention control purposes by the mere presence of a mobile phone (Ward et al., 2017). That is because the same limited resource pool of attentional resources supports both attentional control and other cognitive processes, and resources recruited to suspend automatic attention on the phone become unavailable for other tasks, so performance on those tasks will be significantly affected (Ward et al., 2017). The increasing involvement of these devices in everyday life creates a sense that they are relevant to the goals of their owners, so they are subject to automatic attention (Ward et al., 2017).

In the chronic mismatch between the plethora of environmental information and the limited capacity to process that information, individuals must be selective in allocating attentional resources (Kahneman, 1973; Johnston & Dark, 1986). The priority of a stimulus—the probability of attracting attention—is determined both by its physical “salience” (e.g., location, perceptual contrast) and by the “relevance” of its target (its potential relevance to the target behavior) (Bisley & Mirpour, 2019). Automatic attention generally helps individuals to make the most of their limited cognitive capacity by directing attention to relevant stimuli that are frequently targeted without requiring that these targets be continuously attended to. However, automatic attention can undermine performance when surrounding stimuli are present, such as a mobile phone (Ward et al., 2017). Consistent with this position, research suggests that signals from the phone activate the same system of involuntary attention that responds to the sound of one’s name (Roye et al., 2007). When these devices are salient in the environment, their status as high-priority stimuli suggests that they will exert a gravitational pull on attentional focus. In addition, when individuals are engaged in tasks that their smartphones are not related to, the ability of these devices to automatically attract attention can undermine performance in two ways (Clapp & Gazzaley, 2012). First, smartphones may redirect conscious attention away from the task in focus and toward phone-related thoughts or behaviors. Previous research provides plenty of evidence that individuals spontaneously check their phones during inappropriate times (e.g., Oulasvirta et al., 2012) and that this digital distraction negatively affects performance (End et al., 2009). Second, smartphones may redistribute the attentional resource allocation between engaging in a focal task and suspending attention on the phone.

The results of two experiments performed by Ward et al. (2017) showed that even when users avoid the temptation to check their mobile phones and maintain attention, the mere presence of these devices negatively affects cognitive function, specifically available working memory capacity and functional fluid intelligence. Thornton et al. (2014), who also examined the effect of mobile phone presence on attention, concluded that a visually noticeable mobile phone can negatively affect performance on tasks that require sustained attention. Building on these two seminal papers, a growing number of studies have investigated the potential cognitive effects associated with the mere presence of a smartphone (e.g., Hartanto & Yang, 2016; Hartmann et al., 2020; Pardo & Minda, 2022; Tanil & Yong, 2020). Within this line of research, some studies have repeated these previous findings and confirmed the performance effects associated with smartphone presence, while others have provided null or inconsistent results. The study by Skowronek et al. (2023) showed that in some contexts, the presence of a smartphone may have positive effects.

Mobile phone use has been shown to limit its users’ attention and appropriate timely decision-making, eventually affecting their psychological well-being (Salehan & Negahban, 2013). Altmann et al. (2014) found that interruptions as short as 2.8 s disrupted participants’ concentration flow (several minutes of full, uninterrupted concentration) and increased errors in a serial cognitive task. Thus, smartphones with visual and auditory signals that alert the owner of incoming messages from social networks act as interrupters, disrupting the flow experience and negatively impacting productivity (Duke & Montag, 2017).

### 1.2. Complicated Relationships Between Mobile Phones and Distraction

A decade ago, this state of constant connection would have been unthinkable. Today, it is essential (Ward et al., 2017). Smartphone owners interact with their phones an average of 85 times a day, including as soon as they wake up, just before they go to sleep, and even in the middle of the night (Perlow, 2012; Andrews et al., 2015). Ninety-one percent report never leaving home without their phones, and forty-six percent say they could not live without them (Deutsche Telekom, 2012).

Mobile phone addiction, characterized by excessive and obsessive use of the device, has emerged as a prominent concern in the digital age (Kwon et al., 2013b). People with mobile phone addiction have difficulty disengaging from the device and engage in mobile phone-related activities. This addictive pattern of mobile phone use can lead to attention lapses, reduced cognitive flexibility, and decreased overall well-being (Elhai et al., 2016). Mobile phone addiction distracts individuals from their everyday lives and most of the activities that are part of it (García-Santillán & Espinosa-Ramos, 2021). According to Gill et al. (2012), smartphone use increases reaction time, decreases focus (attention), and lowers the performance of tasks that require cognitive concentration and decision-making. Thomée (2012) suggested that the adverse affective effects of addictive behaviors of smartphone use could arise from three possibilities: (a) the requirement to always be accessible, (b) the nomophobia or fear of losing control of your phone, and (c) distractions caused by smartphones that prevent personal life engagements, such as school or work commitments.

University students seem to be the typical victims of mobile phone addiction. They have strong learning abilities and can adapt to new things (including mobile phones) more quickly, which also brings addiction (Liu et al., 2024). Unfortunately, it was reported that university students were more likely to be vulnerable to MPA (Long et al., 2016). Compared to older social groups, university students are usually mentally immature and less able to self-regulate (Li et al., 2018). Therefore, they are more likely to use mobile phones excessively. Moreover, today’s students are “digital natives” growing up in the mobile phone environment. Therefore, mobile phones have become a necessity in their lives (Long et al., 2016). The forced separation from a ringing telephone can also increase heart rate and anxiety and decrease cognitive performance (Clayton et al., 2015). The increasing prevalence of smartphone dependency (Kwon et al., 2013b) has been linked to heightened feelings of anxiety associated with a lack of smartphone interaction (Gonçalves et al., 2020).

Another aspect of the complex relationship between mobile phones and attention is the phenomenon of nomophobia, or the fear of being without a mobile phone (Yildirim & Correia, 2015). The study identified three key predictors of nomophobia: interpersonal sensitivity, obsessive-compulsive tendencies, and the number of hours spent using smartphones per day (Gonçalves et al., 2020). Consequently, excessive smartphone use can give rise to nomophobia, an acronym derived from the phrase “no mobile” (A. L. S. King et al., 2010). In its most basic form, nomophobia can be defined as the anxiety, discomfort, and stress experienced by an individual when their smartphone is not readily available (A. King et al., 2013). In the context of this study, nomophobia is defined as the fear of being unable to use a smartphone and/or the services it offers. It refers to the fear of being unable to communicate and access information, losing the connectivity that smartphones allow, and giving up the convenience that smartphones provide. It has been proposed that the brain already understands that the smartphone is a “member” of the human body (Jeong et al., 2016). As such, the brain reacts to the absence of smartphone vibration like it does when someone has an amputated arm; they feel it, but it is not there (Kruger & Djerf, 2016). According to Fisoun et al. (2012), there are cases where people already believe and feel that the smartphone vibrated, but this did not happen. In general, nomophobia is a pathological fear of being away from your smartphone and without an internet connection. It can be said that people who have nomophobia feel an unreasonable fear of leaving home without a smartphone and feel great anxiety when they lose it, run out of battery, or have no network coverage. They feel isolated from family and friends because they are not permanently connected to them and, therefore, need to know what others are doing (Bragazzi & Del Puente, 2014). A 2012 study found that women were more likely to be nomophobic and that young adults aged 18–24 years were more susceptible to nomophobia (Securenvoy, 2012). Also, a study conducted in 2022 by Schwaiger and Tahir (2022) among Pakistani students showed that when smartphones were present, they had difficulty with more complex attention tasks despite the level of nomophobia. Considering the rates of mobile phone ownership and social media use, it is unsurprising that nomophobia is prevalent in this population. This is especially applicable among university students who have shown rates of nomophobia of up to 100% (Schwaiger & Tahir, 2022).

While mobile phone addiction and nomophobia share commonalities in terms of their impact on attention, they represent separate but interrelated patterns. Mobile phone addiction is characterized by obsessive and maladaptive patterns of mobile phone use (Y. K. Lee et al., 2014), while nomophobia reflects a fear of not having the device (Securenvoy, 2012). However, the terms phobia and addiction have been recognized alternatively in many studies when introducing the common symptoms of nomophobia (Y. K. Lee et al., 2014; Yildirim & Correia, 2015; A. King et al., 2013; Alosaimi et al., 2016). These studies have suggested that in addition to fear-like symptoms, excessive mobile phone use has been closely linked to the concepts of addiction, compulsion, and anxiety. Some theorists suggest that nomophobia is a misused term and may be better interpreted as a type of anxiety, addiction, or behavioral disorder rather than fear itself (Busch & McCarthy, 2021). Despite the many interpretations, nomophobia is commonly considered a “phobia condition” (Yildirim & Correia, 2015; Lin et al., 2015; A. King et al., 2013; Yildirim, 2014). However, several studies have shown that phobias, addictions, and anxieties are not isomorphic with each other (Y. K. Lee et al., 2014). This may also be because nomophobia is similar to the patterns of personality disorders, but few studies have examined the relationship between nomophobia and other types of mental disorders. In research, there is ideation of nomophobia as a psychopathological obsession, as younger generations seem to be the population most affected by mobile devices to the point that it interferes with aspects of their daily lives (Y. K. Lee et al., 2014; S. Lee et al., 2017; Yildirim & Correia, 2015). Given this context, nomophobia seems to be related to obsession, but the relationship between the two has not been empirically examined (S. Lee et al., 2018).

### 1.3. Aims of the Study

The present study investigated the complex relationship between mobile phone presence, distraction, addiction, nomophobia, and attentional processes in young adults aged 18–25.

The central aim was to elucidate the effects of passive mobile phone presence on attentional control and performance on attentional tasks. We hypothesized that participants exposed to passive mobile phone presence would show lower performance on attentional tasks, meaning that their attention would be distracted by passive mobile phone presence compared to those without mobile phone presence (H1).

We also hypothesized that higher self-reported levels of mobile phone distraction would negatively correlate with attentional performance, leading to increased susceptibility to distraction during task performance (H2).

We also assessed the role of fear of losing one’s phone on attentional processes, hypothesizing that individuals with a higher fear of losing their phone would experience increased distraction when their phone is taken away (H3).

In addition, this study aimed to examine the relationships between self-reported levels of mobile phone distraction, addiction, and attentional performance. We expected that participants who reported higher levels of mobile phone addiction would show lower attentional performance on tasks requiring sustained concentration and cognitive flexibility compared to those with lower levels of addiction (H4).

Finally, in line with the literature, we predicted that women would experience higher levels of nomophobia than men (H5).

## 2. Materials and Methods

### 2.1. Participants

The study sample comprised 144 young adults (75 females and 69 males) aged 18 to 25, with a mean age of 21.5 (SD = 3.0) years. Participants were selected using a snowball sampling technique, where initial participants were recruited based on convenience or specific criteria, i.e., 18 to 25 years old and owning a mobile phone. Then, they referred other potential participants to the study through social media.

The sample size for this study was determined using G*Power software (version 3.1.9.7), assuming a medium effect size (d = 0.5) as suggested by Cohen (1988) and Faul et al. (2007). This choice was made because there were no prior studies with a similar methodological approach to providing an empirical basis for a more specific effect size estimation. The required sample size was calculated using an estimated effect size of d = 0.5, an alpha level of 0.05, and a desired power of 0.80. This calculation is that a sample size of 128 participants would be needed. The final sample consisted of 144 participants, slightly exceeding the minimum requirement and ensuring adequate statistical power.

### 2.2. Materials

Three data collection instruments were employed in this study to assess constructions related to mobile phone usage. A six-point Likert scale (1: “strongly disagree” and 6: “strongly agree”) was utilized for all tools. In the present study, we adapted the response format of some scales to a six-point Likert scale for the sake of consistency across all measures. We acknowledge that this differs from the original five-point format (e.g., 1 = “seldom” to 5 = “almost always”). Notably, we did not conduct a separate validation study for the new response format, nor did we request explicit authorization from the original authors for this modification. While minor alterations to Likert-type scales are sometimes made to improve measurement consistency or sensitivity, we recognize that this approach can constitute a limitation of our research design.

The first author and an independent translator translated and back-translated all questionnaires to ensure linguistic and cultural equivalence. This involved translating the questionnaires from English to Greek and then back-translating them to English to ensure the accuracy and consistency of the translation. Cronbach’s alpha was calculated to assess the internal consistency of each measurement instrument and its subscales. Reliability values for the Smartphone Distraction Scale (SDS) and its subscales, as well as for the Smartphone Addiction Scale-Short Version (SAS-SV) and the Nomophobia Questionnaire (NMP-Q), are reported in Table 1.

The first instrument was the Smartphone Distraction Scale (SDS) (Throuvala et al., 2021), composed of 16 questions and measuring self-reported levels of distraction caused by mobile phone use. The scale consists of 4 subscales (attention impulsiveness, online vigilance, multitasking, and emotion regulation). To confirm the adequacy of our adapted scale, we evaluated its internal consistency and factor structure. Cronbach’s α and McDonald’s ω for the four subscales both ranged from 0.75 to 0.89, and a confirmatory factor analysis indicated good model fit (CFI = 0.91, TLI = 0.90, RMSEA = 0.07), suggesting that the scale remains psychometrically sound despite the modifications.

The second was the Smartphone Addiction Scale-Short Version (SAS-SV) (Kwon et al., 2013a), which consists of 10 questions and assesses individuals’ addictive tendencies toward mobile phones. The scale’s total score ranges from 10 to 60, with cut-off scores of more than 31 in males and more than 33 in females indicating smartphone addiction. In the present study, the tool demonstrated very good internal consistency (α = 0.83).

Finally, the Nomophobia Questionnaire (NMP-Q) (Yildirim & Correia, 2015) consists of 20 questions and measures the fear of being without one’s mobile phone. It also showed good internal consistency (α = 0.94).

The data were analyzed using IBM SPSS Statistics (Version 27). Three participants were excluded from the sample due to being identified as multivariate outliers based on the Mahalanobis distance test. These outliers exceeded the critical value, indicating they had an undue influence on the overall dataset, and were therefore removed to ensure the robustness of the analysis.

The means, standard deviations, and Cronbach’s α reliability coefficients were calculated for each of the instruments in this study. The variables demonstrated sufficient overall consistency and high reliability (α > 0.70). Table 1 below summarizes the values for all variables.

#### The Attention Network Test (ANT)

**Figure 1:**
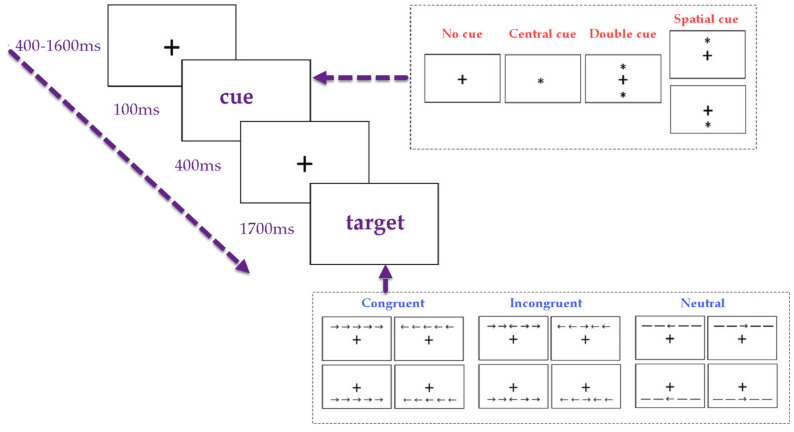
Sequence of events during a trial of the ANT and the cue and target conditions of the test.

The Attention Network Test (ANT) measures attentional control through reaction time and error rates on tasks requiring alerting, orienting, and executive control. The ANT presents different cue types and flanker stimuli, which participants must respond to under varying conditions (Arora et al., 2020). Figure 1 below provides a clear visual representation of the stages involved in a typical ANT trial, including the variations in cue type and target stimuli that participants experience during the task.

Each trial begins with the presentation of a fixation cross (‘+’) at the center of the screen for a variable duration (400–1600 milliseconds) to ensure participant engagement. A cue then appears for 100 milliseconds, with four possible cue conditions:

No Cue: Only the fixation cross is shown, providing no additional visual information.

Central Cue: A single asterisk (*) appears either above or below the fixation cross, signaling the occurrence of a target without specifying its location.

Double Cue: Asterisks appear both above and below the fixation cross, enhancing alertness without indicating the target location.

Spatial Cue: An asterisk appears at the precise location of the upcoming target, guiding spatial attention.

Following cue presentation, the target appears for up to 1700 milliseconds. The target consists of a central arrow that the participant must identify, accompanied by four flanking arrows, which can either facilitate or interfere with decision-making. The target stimuli can take three forms:

Congruent: Flanking arrows point in the same direction as the central arrow, minimizing conflict.

Incongruent: Flanking arrows point in the opposite direction of the central arrow, increasing cognitive load by requiring inhibition of conflicting information.

Neutral: Flanking stimuli are dashes (---), providing no directional influence.

Participants indicate the direction of the central arrow by pressing a corresponding key (left or right). The ANT measures reaction time (milliseconds) and accuracy (correct vs. incorrect responses). The combination of different cue conditions and target configurations allows the test to assess alertness, spatial orientation, and conflict resolution abilities, offering a comprehensive evaluation of attentional networks.

### 2.3. Procedure

The study was approved by the Ethics Committee of the Department of Psychology, National and Kapodistrian University of Athens, on 18 October 2023. Data collection took place from January 2024 to March 2024 in a controlled environment to ensure consistency across all participants. The environment was meticulously regulated to neutralize potential variables such as temperature, noise, and lighting, which might impact the participants’ performance and distraction levels throughout the study.

The real purpose of the research was hidden from all participants to avoid any guidance. Before the start of the study, participants received informed consent information describing the rudimentary purpose of the study, the voluntary nature of participation, and assurances of anonymity and confidentiality of their responses. Participants had to consent by actively agreeing to participate in the study. In addition, participants were informed that no remuneration would be given to them for their participation.

Each participant was tested individually, ensuring confidentiality and reducing the impact of external factors. Participants were similar in terms of their demographics and were therefore randomly allocated to one of two groups: the control group and the experimental group. They were assigned to the experimental vs. control conditions in alternating order upon arrival. This systematic randomization method helped maintain balance across conditions. Participants in the control group were instructed to leave their mobile phones outside the lab to eliminate any potential influence of mobile phone presence.

Conversely, participants in the experimental group were instructed to activate their mobile phones’ data or Wi-Fi functions, set them to vibrate or sound mode, and place them on the computer desk within their line of sight. However, participants were instructed not to touch their mobile phones throughout the experimental task. Once the experimental task had been completed, the participants were informed of the true nature and objectives of the study. This approach helped to ensure the reliability of the data collected, as participants were not influenced and behaved naturally, without any intent to demonstrate that they were unaffected by the presence of their mobile phone. The total duration of the procedure was approximately 30 min, including instructions and debriefing.

## 3. Results

### 3.1. Preliminary Analyses

To ensure the two groups were comparable, we assessed potential baseline differences in age, sex, self-reported feelings when without a phone, and overall mobile phone engagement. None of these comparisons reached statistical significance, suggesting that any observed effects are likely attributable to experimental manipulation rather than pre-existing group differences.

### 3.2. Hypothesis Testing

To examine the effect of passive mobile phone presence on participants’ performance on attentional tasks, two independent sample t-tests were conducted. Descriptive statistics are presented in Table 2.The analyses aimed to test the hypotheses that participants exposed to passive mobile phone presence would show lower performance and more mistakes on attentional tasks compared to those without mobile phone presence (one-tailed hypothesis).

The results showed no significant difference for the ANT time variable [*t*(139) = 0.837, *p* = 0.202, *d* = 0.14] between the two groups. Similarly, for the ANT mistakes variable, there was no statistically significant difference in performance between the two groups [*t*(139) = 1.248, *p* = 0.107, *d* = 0.21]. These findings suggest that the presence of a passive mobile phone does not significantly distract attention or lower performance on attentional tasks compared to conditions without mobile phone presence. The small effect sizes suggest the minimal practical significance of mobile phone presence on attentional task performance. Thus, the hypothesis that passive mobile phone presence would significantly distract attention and lower performance on attentional tasks (H1) was not supported by the data.

To examine the relationships between attentional task performance (time and mistakes) and the study variables under mobile phone absence and presence conditions, Pearson’s correlation coefficients were calculated (see Table 3). Although smartphone distraction (SDS), nomophobia (NMP-Q), and mobile phone addiction (SAS-SV) were strongly and significantly correlated (*r* = 0.66 for distraction and nomophobia, *r* = 0.78 for distraction and addiction, and *r* = 0.67 for nomophobia and addiction, all *p* < 0.001), relationships between these variables and attentional performance were generally weak.

Notably, SDS Attention Impulsiveness and SAS-SV showed a significant positive correlation with the number of mistakes made during the attentional task when the mobile phone was present, suggesting that higher levels of impulsiveness and smartphone addiction are associated with more errors in the presence of a phone. Other correlations were weak and did not reach significance. Interestingly, SDS Multitasking and SAS-SV were negatively correlated with task time when the mobile phone was present (*r* = −0.15 in both cases), implying that frequent multitaskers and those with higher levels of smartphone addiction tended to complete the task faster in the presence of a phone, though these correlations were not statistically significant. Overall, the findings suggest that the presence of a mobile phone may impact attentional performance, particularly among individuals with higher smartphone addiction (H2).

Contrary to the third hypothesis, which proposed that individuals with a higher fear of losing their phone would experience increased distraction when their phone is absent, the findings presented in Table 3 did not provide support for this claim. Although a non-significant positive correlation (*r* = 0.20) was observed between fear of losing the phone (NMP-Q) and ANT errors when the phone was present, this relationship was not statistically significant, indicating limited evidence for the predicted effect.

**Figure 2:**
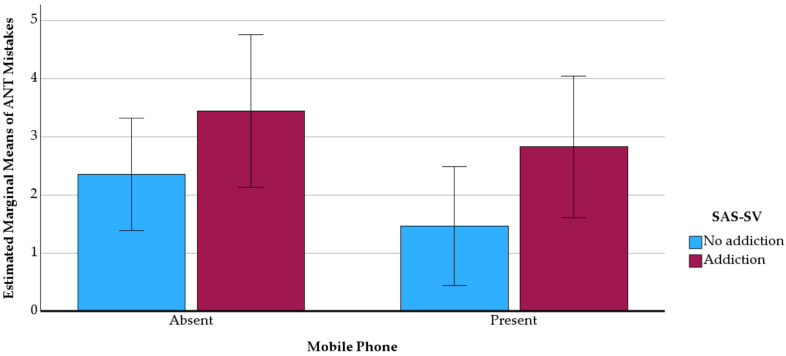
Interaction of mobile phone presence and mobile phone addiction on attentional task performance (errors).

A two-way between-groups ANOVA was conducted to investigate the effects of self-reported mobile phone addiction and mobile phone presence on attentional performance, measured by errors and completion time in the ANT. Participants were classified into two groups based on their SAS-SV scores: no addiction (score ≤ 32, *n* = 87) and addiction (score ≥ 33, *n* = 54). The analysis revealed a significant main effect of addiction level on errors [*F*(1, 137) = 4.548, *p* = 0.035, *η^2^_p_* = 0.032], indicating that individuals with higher levels of addiction made more mistakes. However, no significant interaction was found between mobile phone addiction and phone presence (see Figure 2). For completion time, neither the main effects nor the interaction reached statistical significance. Thus, the hypothesis (H4) that higher levels of SAS-SV would be associated with poorer attentional performance on tasks requiring sustained concentration and cognitive flexibility was only partially supported, as depicted in Figure 2.

Finally, an independent samples t-test was performed to assess differences in nomophobia levels between women and men. It was hypothesized that women would report higher levels of nomophobia than men (H5). The results revealed a statistically significant difference in nomophobia scores between women and men [*t*(139) = 6.175, *p* < 0.001, *d* = 1.04]. The mean difference of 18.7 points (78.5 vs. 59.8) on the nomophobia scale, along with a large effect size, indicates a substantial gender difference in the experience of nomophobia. These findings support the hypothesis that women experience higher levels of nomophobia than men.

## 4. Discussion

The objective of the present study was to examine the impact of mobile phone presence on attentional processes. This was performed by investigating phenomena such as mobile phone addiction and nomophobia. Both of these have been identified as factors that can complicate the relationship with mobile phones. The study was guided by the hypothesis that the mere passive presence of mobile phones might potentially act as a distraction for individuals, resulting in reduced performance on attention-demanding activities. This hypothesis was investigated through a series of tests, each aimed at identifying different aspects of the relationship between the presence of mobile phones and the ability to regulate attention.

The findings of this study present a complex picture that is both aligned with and distinct from literature. In particular, the hypothesis that the passive presence of a mobile phone would significantly distract and impair attentional performance (H1) was not supported by the data. This finding is noteworthy and contrary to the prevailing view that the passive presence of mobile phones has a detrimental effect on cognition (Thornton et al., 2014; Ward et al., 2017). One potential explanation for this discrepancy is the differing methodologies and definitions of distraction employed in the various studies. Previous studies have primarily focused on active phone use or immediate response to notifications (Thornton et al., 2014; Ward et al., 2017). This study, however, examines the simple presence of the mobile phone in the room, which may not have as significant an impact on attentional control.

Indeed, mobile phones are evolving from smartphones to cognitive phones, which can understand our lifestyles, health, and well-being; help us navigate our days; and intervene on our behalf (Campbell & Choudhury, 2012). An alternative hypothesis is that individuals have adapted to the pervasive presence of mobile phones in their environment, thereby reducing the impact of these devices on attentional tasks. This follows the findings of Ophir et al. (2009), who demonstrate that individuals can exert cognitive control in environments requiring multitasking and develop parallel information processing strategies, potentially offsetting the distracting effects of mobile phone presence on attention tasks.

One possible explanation for the absence of significant effects is the habituation hypothesis, which suggests that individuals have adapted to the ubiquitous presence of mobile phones, reducing their potential for distraction. Prior research has demonstrated that frequent exposure to a stimulus can lead to cognitive adaptation, diminishing its impact over time (Wiradhany & Nieuwenstein, 2017). Given the high daily exposure of young adults to mobile devices, their attentional systems may have adjusted to their presence, allowing them to maintain focus even in their vicinity.

Additionally, the cognitive demands of the attentional task used in this study may not have been high enough to elicit significant differences between conditions. Prior research suggests that the distracting effects of mobile phones are more pronounced in high-demand tasks that require sustained attention and executive control (Hartmann et al., 2020). It is possible that the experimental task used in this study did not generate sufficient cognitive load to reveal such differences. Future research could explore whether increasing task difficulty amplifies the potential impact of mobile phone presence on attentional performance.

Furthermore, the absence of a significant correlation between self-reported levels of mobile phone distraction and performance on attention tasks (H2) provides further insight into the discussion. This suggests that individuals’ perceptions of distraction do not necessarily result in the observed and measurable deficits in attentional performance that might otherwise be expected. The absence of a correlation between self-reported mobile phone distraction and actual attentional performance indicates the existence of a discrepancy between perception and action in digital behavior. Individuals may overestimate how much mobile phone distraction affects their cognitive abilities due to heightened awareness of their digital behavior. Individuals with higher metacognitive abilities can effectively monitor and regulate their attentional focus independently of subjective perceptions of distraction (Nelson, 1990). Metacognitive awareness, which includes knowledge of and regulation of knowledge, is multidimensional, general, and teachable (Zepeda et al., 2015). It is also possible that individuals have developed coping mechanisms or that factors beyond self-reported distraction, such as work engagement or cognitive load, influence attentional control. Increased cognitive load may lead to increased concentration and engagement with the primary task, suppressing the processing of irrelevant stimuli and thereby reducing the ability to distract (Sörqvist et al., 2016). A high perceptual load reduces distractor interference, whereas a low cognitive load increases distractor interference in selective attention. The possible discrepancy between subjective experiences of distraction and objective attentional outcomes highlights the complexity of attention as a cognitive process that allows individuals to focus on goals rather than distractions (Burgoyne & Engle, 2020) and the multifaceted nature of distraction that is influenced by task complexity (Graydon & Eysenck, 1989).

The present study found only one significant—but weak—relationship between levels of mobile phone dependence and performance on attentional tasks (H4). This unexpected finding challenges traditional understandings of the harms associated with addiction and the assumption that excessive mobile phone use inevitably leads to attention deficits. Rather than directly affecting attentional control, mobile phone addiction may manifest as a form of habitual behavior distinct from cognitive deficits, and individuals may retain functional abilities despite addictive behavior. It is also likely to be explained by the possibility that individuals with higher levels of addiction may have developed compensatory strategies to maintain attentional control, or other variables not measured in the present study may affect attentional performance. Attentional bias, reward processing, and error processing are neurocognitive mechanisms involved in addiction (Franken & van de Wetering, 2015) and are influenced by a variety of variables. These findings have again brought to the surface the question of whether there is a cell phone addiction in the digital age. Various researchers have expressed their doubts about mobile phone addiction and suggested the terms problematic or maladaptive mobile phone use (Panova & Carbonell, 2018). Other researchers suggest that mobile phone addiction is prevalent among young people and is related to personality traits, sleep disorders, anxiety, stress, and substance use (De-Sola Gutiérrez et al., 2016). Variables such as personality traits or sleep disorders may have contributed to different effects on distraction. Rewards also play a critical role in controlling attention, with reward-related stimuli automatically capturing attention even when they are unobtrusive and task-irrelevant, highlighting a value-based approach to information processing (Anderson, 2016). A previous study among medical students found a high prevalence of smartphone addiction. However, there was no statistically significant correlation between smartphone addiction and attention, suggesting that the relationship may be more complex and not directly causal (Prasetyadi & Gustaman, 2022). The findings certainly highlight the need for a more nuanced understanding of mobile phone addiction and its effects on cognition.

Another relevant aspect to consider is the relationship between excessive smartphone use, multitasking capability, and attention regulation. Modern digital environments frequently demand high levels of multitasking, with individuals constantly switching between tasks, notifications, and digital interactions. While some studies suggest that habitual multitasking may enhance task-switching efficiency, others indicate that excessive engagement with multiple streams of information can lead to diminished sustained attention and increased susceptibility to distractions (Wiradhany & Nieuwenstein, 2017; Cain et al., 2016). Furthermore, excessive smartphone use has been linked to potential alterations in learning processes, as continuous exposure to fragmented information may reduce deep learning and comprehension (Rosen et al., 2013). Future research should explore whether smartphone multitasking might train individuals to operate in high-stimulation environments at the cost of focused, undivided attention.

Investigating the effects of nomophobia on attentional performance did not reveal particularly noteworthy findings. While nomophobia correlated with self-reported mobile phone distraction significantly, it had no measurable effect on attentional performance (H3). People who reported higher fear of losing their phone also reported higher levels of distraction. This finding is consistent with previous research suggesting that fear of being without a mobile phone may be a factor in increased vigilance and distraction: anxiety impairs the efficient functioning of the target attention system and increases stimulus-driven attention but may not impair performance efficiency if it leads to compensatory strategies (Eysenck & Derakshan, 2011). The cognitive and affective aspects of mobile phone-related concerns may not directly affect attentional control when performing procedures in the presence of a mobile phone. The psychological effects of mobile phones go beyond direct cognitive effects, and further research is needed to understand and include broader emotional and behavioral dimensions.

The clear difference in nomophobia scores between genders confirms hypothesis (H5) and is supported by the existing literature, which indicates that women may experience higher levels of nomophobia than men (Securenvoy, 2012). This gender difference highlights the importance of considering demographic variables in understanding the psychological impact of mobile phone use. Understanding these gender differences in nomophobia is important for developing targeted interventions and support strategies to mitigate the negative consequences of mobile phone use.

### Limitations and Future Research Proposals

This study, which sought to adopt a comprehensive approach to understanding the impact of mobile phone presence on attentional processes, has its limitations. Methodologically, snowball sampling may result in a selection bias due to the convenience and networks of the initial participants, which could limit the generalizability of the findings. Furthermore, although sufficient for the analyses conducted, the sample size may not fully encompass the full spectrum of variability in the broader population. It would be beneficial for future studies to utilize a larger and more diverse sample to enhance the external validity of the results. Although the experimental environment was controlled for external factors such as lighting and noise, individual differences (e.g., cognitive abilities, fatigue levels, and prior smartphone usage habits) were not specifically accounted for. Future studies may consider incorporating these factors to better understand their impact on attentional performance.

While the psychometric instruments used in this study, including the Smartphone Distraction Scale, the Smartphone Addiction Scale-Short Version, and the Nomophobia Questionnaire, demonstrated satisfactory reliability, they may be limited in their ability to capture the complex nature of mobile phone-related behaviors and attitudes. In addition, the Attention Network Test, used to measure attentional control, may not encompass all aspects of attention affected by mobile phone presence. Further research could explore additional or alternative measures to provide a more nuanced understanding of these constructions. Another limitation relates to the modification of certain scales from five-point to six-point Likert response formats without conducting an independent validation process. In addition, we did not obtain explicit permission from the original scale authors for this change. Although such adjustments are often performed to unify measurement formats or increase scale sensitivity, ideally, a formal validation or pilot test should be carried out to ensure that reliability and validity are not compromised. We recommend that future studies intending to adapt established scales follow a more rigorous validation procedure and, whenever feasible, seek authorization from the original authors.

In light of the findings and limitations of this study, several avenues for further research are proposed. These findings contribute to the growing body of literature on the impact of mobile phone usage on cognitive functions, suggesting that while mobile phones are an integral part of modern life, their effect on cognitive abilities, such as attention, might be more nuanced than previously assumed. Consequently, further research is required to understand the underlying mechanisms that drive these relationships. Future studies could employ a longitudinal design, experimental methodology, and intervention approach to explore the long-term effects of mobile phone usage on cognitive functioning and to develop strategies to mitigate potential negative impacts. By addressing these research gaps, we can inform the development of interventions and policies to promote healthy smartphone habits and support cognitive well-being in an increasingly digital world.

Future studies could explore the long-term effects of mobile phone presence on attentional control and cognitive performance, considering chronic exposure to mobile phones in daily life. Longitudinal studies would allow for the examination of temporal relationships and potential causal pathways between variables over time. Additionally, research could investigate the potential moderating and mediating factors influencing the relationship between mobile phone use and attention, such as personality traits, coping strategies, and social support. Investigating the impact of mobile phone presence in real-world settings, such as classrooms or workplaces, could provide insights into the practical implications of these findings. Further insight into the circumstances under which mobile phones become a significant source of distraction could be gained through experimental studies that manipulate the salience and relevance of mobile phones. Intervention studies could evaluate strategies to mitigate the adverse effects of mobile phone presence on attentional processes and psychological well-being. For example, mindfulness-based interventions or digital detox programs could be implemented and evaluated for their impact on attentional control and mobile phone-related behaviors. Innovations in technology, such as smartphone applications or wearable devices, could be developed to monitor and manage mobile phone usage to improve the regulation of attention and cognition. Such developments may be facilitated by collaborations between researchers and technology developers, resulting in tools that promote healthy smartphone habits and support cognitive functioning.

The current study’s findings have implications for educational and occupational contexts where attentional control is critical. Therefore, it is recommended that educators and employers consider policies or interventions to minimize the negative effects of mobile phone-related distractions on cognitive performance. Creating educational programs designed to raise awareness of the potential cognitive costs associated with mobile phone usage in attention-demanding situations represents an avenue for future research. This would enable individuals to make more informed decisions about their use of mobile technology.

In conclusion, although this study has advanced the understanding of mobile technology’s impact on attentional processes, further research is required in this field. The identification and resolution of the study’s limitations, together with a more comprehensive understanding of the proposals outlined, will contribute further to the body of knowledge on mobile technology’s cognitive and psychological effects.

## 5. Conclusions

In conclusion, this study sought to investigate the impact of mobile phone presence on attentional processes among young adults, a demographic that is highly engaged with digital technology. The findings provide valuable insights into the intricate relationship between mobile phone use and cognitive functioning. Although the passive presence of mobile phones did not significantly distract attention or impair performance on attentional tasks, self-reported levels of distraction, addiction, and nomophobia were found to be associated with subjective experiences of mobile phone-related concerns regarding mobile phone use. These results align with recent research suggesting that the psychological impact of mobile phones may be more dependent on individual differences rather than their mere presence (Ward et al., 2017; Hartmann et al., 2020). Furthermore, gender differences were observed in nomophobia, with women reporting higher levels than men.

These findings emphasize the need to account for individual differences when assessing the effects of mobile phone presence on attention. Rather than enforcing generalized restrictions, a more tailored approach may be more effective in minimizing distractions in educational and occupational settings. Future research should further explore the long-term cognitive consequences of habitual smartphone use, utilizing longitudinal and real-world experimental designs to provide deeper insights into its effects. Additionally, exploring the moderating role of task complexity and cognitive load could further clarify the conditions under which mobile phones affect attentional performance. By addressing these gaps, researchers can better inform strategies to manage digital distractions and support cognitive well-being in an increasingly connected world.

## Figures and Tables

**Table 1:** Descriptive statistical measures and Cronbach’s alpha coefficients of the variables under study for the total sample (*n* = 141).

	*Mean*	*SD*	*Alpha*
SDS	53.8	13.9	0.88
Attention Impulsiveness	14.9	4.3	0.80
Online Vigilance	9.8	4.1	0.77
Multitasking	15.0	4.4	0.74
Emotion Regulation	14.2	5.1	0.81
SAS-SV	30.0	9.6	0.83
NMP-Q	69.4	20.2	0.94
ANT time (in secs)	23,208.9	5992.7	-
ANT mistakes	2.4	3.4	-

**Table 2:** Descriptive statistics for attentional task performance without and with mobile phone presence.

	Group	*n*	Mean	SD
ANT time (in secs)	Mobile Phone Absent	71	23,628.8	6181.0
	Mobile Phone Present	70	22,783.1	5808.8
ANT mistakes	Mobile Phone Absent	71	2.7	3.8
	Mobile Phone Present	70	2.0	2.8

**Table 3:** Pearson correlation coefficients between attentional task performance (with and without mobile phone) and study variables.

	Mobile Phone Absent		Mobile Phone Present	
	ANT Time	ANT Mistakes	ANT Time	ANT Mistakes
SDS	0.00	0.02	−0.07	0.22
Attention Impulsiveness	0.07	−0.04	−0.05	0.25 *
Online Vigilance	−0.07	−0.07	−0.06	0.16
Multitasking	0.04	0.00	−0.15	0.23
Emotion Regulation	−0.03	0.13	0.05	−0.06
NMP-Q	−0.03	−0.06	−0.11	0.20
SAS-SV	−0.01	0.11	−0.15	0.37 **

* *p* < 0.05; ** *p* < 0.01.

## Data Availability

The raw data supporting the conclusions of this article will be made available by the authors upon request.
